# Limb Salvage After Chronic Nonunion Following Deformity Correction in Congenital Fibular Deficiency: A Cautionary 12-Year Follow-Up Case Highlighting Biological and Mechanical Reconstructive Challenges

**DOI:** 10.3390/clinpract16070125

**Published:** 2026-07-03

**Authors:** Koji Nozaka, Shohei Murata, Naohisa Miyakoshi

**Affiliations:** Department of Orthopaedic Surgery, Akita University Graduate School of Medicine, 1-1-1 Hondo, Akita 010-8543, Japan

**Keywords:** fibular hemimelia, congenital fibular deficiency, nonunion, Ilizarov, distraction osteogenesis, limb salvage, LIPUS

## Abstract

**Background**: Congenital fibular deficiency is a rare longitudinal deficiency of the lower extremity associated with limb-length discrepancy, ankle and foot deformity, soft-tissue imbalance, and functional impairment. Reconstruction may be challenging because bone healing, regenerate maturation, and mechanical stability can be less predictable in selected patients. **Case Presentation**: A man with congenital fibular deficiency developed chronic distal tibial nonunion after corrective osteotomy at another institution. The nonunion persisted for four years, and the patient presented to our hospital with inability to bear weight and wheelchair dependence. A comprehensive salvage strategy was performed, including Achilles tendon lengthening using the Vulpius technique, removal of retained fixation material, debridement and refreshment of the nonunion site, negative bacteriological cultures, autologous cancellous iliac bone grafting, acute shortening and compression of the docking site, circular external fixation, proximal tibial osteotomy, and gradual lengthening. Low-intensity pulsed ultrasound was applied postoperatively as an adjunctive biological stimulus. **Results**: Bone union was achieved, and the external fixator was removed approximately one year after surgery. A total lengthening of 78 mm was achieved. At 12-year follow-up, the AOFAS ankle-hindfoot score was 90, ankle range of motion was 5° dorsiflexion and 40° plantarflexion, and the JOA knee score was 95. The patient walked independently without assistive devices and continued to work. Mild residual varus deformity of the proximal tibia was present, but the patient reported no knee pain, ankle pain, or ankle instability, and radiographs showed no progressive osteoarthritic changes. **Conclusions**: In selected patients with congenital fibular deficiency and chronic nonunion after previous treatment, durable limb salvage may be achieved using an individualized strategy that addresses both biological and mechanical factors.

## 1. Introduction

Congenital fibular deficiency, also referred to as fibular hemimelia, is a rare longitudinal deficiency of the lower extremity with a broad spectrum of musculoskeletal abnormalities [1,2,3,4,5,6]. Patients may present with limb-length discrepancy, ankle valgus deformity, foot deformity, ankle instability, equinus contracture, soft-tissue imbalance, and gait disturbance [3,4,5,6]. These deformities can impair mobility, weight-bearing ability, footwear use, participation in daily activities, and long-term function. The general treatment goals are to obtain a functional plantigrade foot, restore stable weight-bearing, improve limb alignment, and address limb-length discrepancy.

Treatment options vary according to deformity severity, predicted limb-length discrepancy, foot reconstructability, joint stability, patient age, functional demands, and patient preference. Historically, amputation procedures such as Syme or Boyd amputation have been considered in severe cases, whereas limb reconstruction may include deformity correction, soft-tissue balancing, circular external fixation, and distraction osteogenesis [5,6]. Modern reconstructive strategies emphasize that management should address not only limb length and alignment but also ankle stability, foot position, soft-tissue balance, and long-term function [5,6].

Circular external fixation based on Ilizarov principles allows gradual deformity correction, stable multiplanar fixation, early weight-bearing, and limb lengthening [1,2,7,8]. However, previous studies have reported delayed consolidation, regenerate abnormalities, refracture, recurrent deformity, and prolonged treatment courses during limb reconstruction for fibular hemimelia [6,9]. These observations suggest that surgical planning may benefit from consideration of both mechanical alignment and potential biological challenges associated with reconstruction, particularly in complex cases or those with previous treatment failure.

We report a cautionary adult case of congenital fibular deficiency with chronic distal tibial nonunion after prior deformity correction. The purpose of this case report is to highlight the importance of careful diagnosis, individualized reconstructive planning, and simultaneous management of biological and mechanical problems in limb salvage after failed prior treatment.

## 2. Case Presentation

### 2.1. Patient Information

A 35-year-old man with congenital fibular deficiency was referred to our institution because of chronic distal tibial nonunion following deformity correction performed at another hospital. The patient was 160 cm tall and weighed 95 kg (body mass index, 37.1 kg/m^2^). He had completed high school education and worked in a family-owned retail business with his parents.

According to the patient, he had been aware of mild ankle valgus deformity since childhood. Although he had visited orthopedic clinics intermittently during childhood and adolescence, congenital fibular deficiency had not been formally explained to him. Despite the deformity, he remained ambulatory during adolescence and adulthood and was able to participate in daily activities. In his twenties, progressive ankle pain developed and gradually interfered with walking. He subsequently consulted another institution, where corrective surgery was performed for ankle deformity at 31 years of age.

### 2.2. Clinical Findings

**Figure 1:**
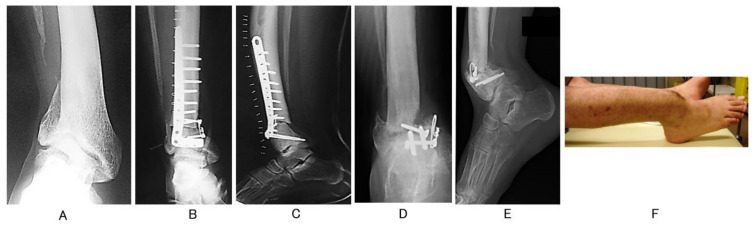
Clinical course prior to referral. (**A**) Preoperative anteroposterior radiograph showing ankle deformity associated with congenital fibular deficiency. (**B**,**C**) Anteroposterior and lateral radiographs immediately after deformity correction osteotomy performed at the previous institution. (**D**,**E**) Anteroposterior and lateral radiographs demonstrating nonunion at the osteotomy site with broken screws. (**F**) Clinical photograph at presentation showing anterior angulation at the nonunion site.

Following corrective surgery at another institution, nonunion developed at the distal tibial osteotomy site. The initial procedure consisted of a distal tibial closed-wedge osteotomy performed through an anterolateral approach. Fixation was achieved using an anterolateral plate and supplemental medial cerclage wiring (Figure 1B,C). Postoperatively, cast immobilization and non-weight-bearing were maintained for 4 weeks, followed by progressive weight-bearing, with full weight-bearing initiated at 8 weeks.

Eight months after surgery, implant failure occurred with plate breakage at the osteotomy site, resulting in established nonunion (Figure 1D,E). The plate was subsequently removed; however, the broken screws and medial cerclage wiring were retained in situ. No further reconstructive procedure for the nonunion was undertaken thereafter. The condition remained untreated for four years despite consultations at multiple institutions. The patient subsequently presented to our institution after further reconstruction was considered difficult at the previous institution.

At presentation to our hospital, the patient complained of inability to walk and instability of the lower leg. He was wheelchair-dependent and unable to bear weight on the affected limb. Clinical examination revealed marked instability at the distal tibial nonunion site with anterior angulation (Figure 1F). Although ankle valgus deformity and ankle pain had been present before the initial surgery, the dominant clinical problem at referral was longstanding nonunion with painful instability and loss of weight-bearing ability rather than ankle deformity alone.

### 2.3. Timeline

- Childhood to adolescence: Mild ankle valgus deformity was recognized, but the patient remained ambulatory.
- Adulthood: Progressive ankle pain developed and interfered with walking.
- Age 31: Corrective osteotomy was performed at another institution.
- 8 months after initial surgery: Implant failure and distal tibial nonunion developed.
- Following 4 years: Persistent tibial nonunion and progressive loss of weight-bearing ability.
- Age 35: Referred to our institution with wheelchair dependence.
- Approximately 1 year postoperatively: Bone union was achieved and the circular external fixator was removed.
- 12 years postoperatively: Final follow-up.

### 2.4. Diagnostic Assessment

Preoperative radiographs at the previous institution demonstrated ankle deformity associated with congenital fibular deficiency (Figure 1A). Radiographs obtained immediately after deformity correction osteotomy at the previous institution are shown in Figure 1B,C. Subsequent imaging revealed nonunion at the osteotomy site with broken screws (Figure 1D,E). Clinically, anterior angulation and instability were evident at presentation to our institution (Figure 1F).

There were no clinical findings suggesting active infection, such as a draining sinus, local erythema, purulence, or fever. Intraoperative cultures were obtained as described below.

### 2.5. Therapeutic Intervention

A comprehensive reconstructive strategy was planned to address equinus contracture, the chronic nonunion site, mechanical instability, limb alignment, and limb-length discrepancy.

The patient was positioned supine. Because marked equinus contracture was present, Achilles tendon lengthening was first performed using the Vulpius technique with the affected limb placed in a cross-legged position. Before the procedure, maximum ankle dorsiflexion with the knee extended was approximately −30°. After the Vulpius procedure, dorsiflexion improved to approximately +10°.

The distal tibial nonunion site was then exposed through an anterior approach. The retained fixation material was removed under direct visualization as much as feasible. Intraoperative tissue specimens obtained from the nonunion site were submitted for bacteriological examination; all cultures were negative. Fibrous tissue was removed, and the nonunion site was thoroughly debrided and refreshed. Removal of the broken screws created substantial bone defects, which were filled with autologous cancellous bone graft harvested from the iliac crest.

**Figure 2:**
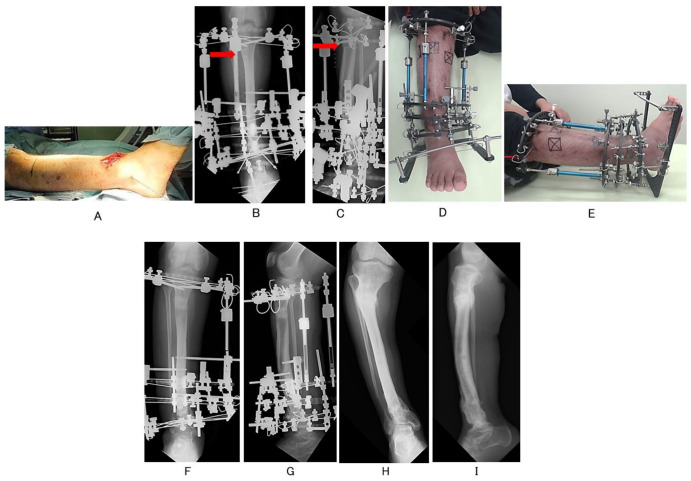
Surgical procedure and postoperative course at our institution. (**A**) Intraoperative photograph showing exposure and refreshment of the distal tibial nonunion site after removal of retained fixation material. (**B**,**C**) Immediate postoperative anteroposterior and lateral radiographs. Red arrows indicate the proximal osteotomy site for distraction osteogenesis. (**D**,**E**) Clinical photographs during the distraction phase. (**F**,**G**) Anteroposterior and lateral radiographs at 9 months postoperatively, showing regenerate formation after completion of lengthening. (**H**,**I**) Anteroposterior and lateral radiographs at the latest follow-up, demonstrating bony continuity of the docking site and distraction regenerate, mild residual varus deformity of the proximal tibia, and durable limb preservation.

The distal fragment demonstrated cup-shaped morphology and poor local bone quality. After chipping and preparation of the nonunion surfaces, approximately 14 mm of acute shortening was performed at the docking site. This allowed stable compression of the nonunion site with the ankle maintained in a plantigrade position. A circular external fixator based on the Ilizarov method was then applied. Multiple wires were inserted into the distal fragment to maximize fixation stability despite the small dysplastic bone segment. Immediate postoperative radiographs are shown in Figure 2B,C.

Subsequently, a proximal tibial osteotomy was performed for distraction osteogenesis. Gradual lengthening was performed at a rate of 1 mm per day, divided into four increments (0.25 mm × 4). The distraction period was approximately 78 days, and a total lengthening of 78 mm was achieved. Clinical photographs during the distraction phase are shown in Figure 2D,E.

Low-intensity pulsed ultrasound (LIPUS) therapy (Exogen®, Bioventus, Durham, NC, USA) was initiated immediately after surgery and applied to both the nonunion site and the distraction regenerate segment. The patient performed LIPUS treatment once daily for 20 min, and therapy was continued for approximately 12 months postoperatively.

Throughout the distraction and consolidation phases, supervised rehabilitation emphasized full weight-bearing gait training with the circular external fixator in place. The frame was intentionally maintained until sufficient maturation of the regenerate and consolidation of the nonunion site were confirmed radiographically. Radiographs at 9 months postoperatively, showing regenerate formation, are shown in Figure 2F,G. The healing index was 41.3 days/cm.

### 2.6. Postoperative Management

After surgery, the patient underwent full weight-bearing gait training during the distraction and consolidation phases while the circular external fixator was in place. Active and passive range-of-motion exercises of the knee and ankle were encouraged as tolerated. After removal of the external fixator, a patellar tendon-bearing (PTB) orthosis was used for approximately 2 months, and gait training was continued with progressive transition to unassisted ambulation.

### 2.7. Follow-Up and Outcomes

Preoperatively, no standardized functional scores such as the AOFAS ankle-hindfoot score or JOA knee score were available because the patient had been wheelchair-dependent for several years before referral. Therefore, quantitative comparison between preoperative and postoperative functional scores was not possible. Functionally, the baseline status at referral was characterized by inability to bear weight, marked instability at the distal tibial nonunion site, and wheelchair dependence.

Bone union was achieved approximately one year after surgery, and the external fixator was removed. The patient regained weight-bearing ability for the first time in five years. At the latest follow-up, 12 years after surgery, radiographs demonstrated bony continuity of both the docking site and the distraction regenerate without evidence of recurrent nonunion or refracture (Figure 2H,I). Mild residual varus deformity was present in the proximal tibia.

**Figure 3:**
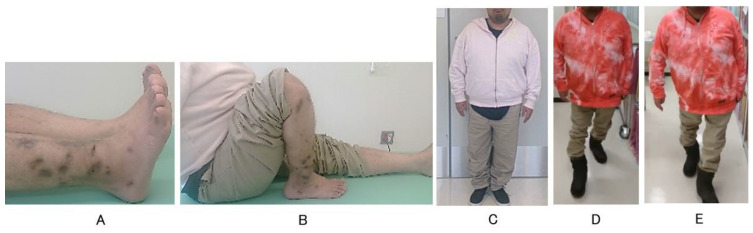
Functional outcome at 12 years postoperatively. (**A**) Ankle dorsiflexion. (**B**) Full knee flexion. (**C**) Standing posture showing a plantigrade foot with mild residual proximal tibial varus alignment. (**D**,**E**) Walking without a cane, demonstrating independent ambulation with stable weight-bearing.

Functionally, the AOFAS ankle-hindfoot score was 90 points [10], ankle range of motion was 5° in dorsiflexion and 40° in plantarflexion, and the JOA knee score was 95 points, with knee range of motion from 0° to 140° (Figure 3A,B). The patient reported no knee pain, no ankle pain, and no ankle instability. Radiographs showed no progressive osteoarthritic changes in either the knee or ankle joint. He was able to ambulate independently without assistive devices, continued to work in the family-owned retail business, and maintained independent daily activities. Standing posture demonstrated a plantigrade foot (Figure 3C), and independent walking with stable weight-bearing was observed (Figure 3D,E).

## 3. Discussion

The present case illustrates that congenital fibular deficiency may present reconstructive challenges beyond correction of deformity alone. Previous studies have reported delayed consolidation, regenerate abnormalities, refracture, recurrent deformity, and prolonged treatment courses during limb reconstruction in patients with fibular hemimelia [1,2,3,4,5,6,7,8,9]. These observations suggest that, in some patients, bone healing and regenerate maturation may be less predictable than expected during reconstruction.

In the present case, the prior surgery corrected deformity but was followed by chronic nonunion and prolonged loss of weight-bearing function. This clinical course highlights a potential pitfall in limb reconstruction: strategies focused primarily on alignment restoration may be insufficient in selected patients with congenital fibular deficiency, particularly when local bone quality, mechanical stability, soft-tissue balance, and limb-length discrepancy are not adequately addressed. The underlying challenge may involve a complex interplay of bone morphology, mechanical instability, abnormal loading, soft-tissue imbalance, and limb-length discrepancy rather than alignment alone.

Comparison with previously published reports is important when interpreting the present case. Mishima et al. reported delayed consolidation, regenerate fracture, and other complications during limb lengthening for fibular hemimelia, highlighting the complexity of reconstruction in these patients [9]. Similarly, Fuller et al. emphasized that successful reconstruction requires careful management of deformity, limb-length discrepancy, ankle stability, and soft-tissue balance rather than simple correction of alignment alone [6]. The present case is consistent with these observations and further suggests that limb salvage may still be possible after failed prior treatment and longstanding nonunion when both biological and mechanical factors are addressed comprehensively.

Alternative treatment strategies should also be considered. In severe congenital fibular deficiency, amputation procedures such as Syme or Boyd amputation have historically been advocated as treatment options because of concerns regarding prolonged treatment, multiple procedures, and reconstruction-related complications [11]. Internal fixation with corrective osteotomy may also be appropriate in selected patients when adequate bone quality, stable fixation, and predictable healing can be expected. However, in the present case, longstanding nonunion, retained hardware, poor local bone conditions, deformity, and loss of ambulatory function made biological reconstruction with circular external fixation and distraction osteogenesis a reasonable salvage strategy. The choice of treatment should therefore be individualized according to the severity of deformity, bone condition, functional status, patient preference, and feasibility of prolonged reconstruction.

The salvage strategy used in this case was designed to address both biological and mechanical insufficiency. Refreshment of the nonunion site combined with autologous cancellous iliac bone grafting was performed to enhance local osteogenic potential. Acute shortening improved bony apposition, increased local stability, reduced soft-tissue tension, and enabled restoration of a plantigrade foot. Circular external fixation provided stable multiplanar fixation without relying on a single internal implant. In this context, the external fixator functioned not only as a fixation device but also as a reconstructive platform capable of simultaneously managing alignment, stability, and limb length [1,2].

Another important aspect of this case is the combination of docking-site reconstruction and proximal tibial distraction osteogenesis. Following acute shortening and stabilization of the nonunion site, gradual lengthening was performed to compensate for shortening and limb-length discrepancy. The distraction protocol was based on classic Ilizarov principles, which emphasize gradual lengthening at an appropriate rate and frequency to optimize regenerate formation [7,8]. In the present case, prolonged protected fixation and weight-bearing during the frame period were intended to support both mechanical stability and regenerate maturation.

LIPUS was applied as an adjunctive biological stimulus to both the nonunion site and the distraction regenerate. However, the clinical evidence for LIPUS remains heterogeneous, and its independent efficacy is still debated [12,13,14]. Therefore, the favorable outcome in this case should not be attributed to LIPUS alone. Rather, LIPUS should be interpreted as one component of a broader reconstructive strategy that included nonunion-site refreshment, autologous cancellous bone grafting, stable circular external fixation, distraction osteogenesis, and prolonged mechanical loading.

Despite the complexity and complication profile reported in previous reconstruction series [6,9], the present case demonstrated durable limb preservation and functional recovery after salvage of longstanding nonunion. Mild residual varus deformity of the proximal tibia was present at long-term follow-up; however, the patient reported no knee pain, ankle pain, or ankle instability, and radiographs showed no progressive osteoarthritic change in the knee or ankle. This outcome should be interpreted cautiously as a durable result in a selected case rather than as comparative evidence of superiority over other reconstructive strategies.

This case also provides an educational implication. In congenital fibular deficiency, reconstruction may require consideration of factors beyond deformity correction alone, including local bone quality, mechanical stability, soft-tissue balance, and limb-length discrepancy. Although conclusions cannot be drawn from a single case, the present experience suggests that insufficient attention to these factors may contribute to persistent nonunion despite apparent alignment correction.

## 4. Limitations

This study has several limitations. First, it represents a single case report, and therefore the findings cannot be generalized to all patients with congenital fibular deficiency. Second, although long-term functional recovery was favorable, preoperative standardized functional scores and internationally standardized patient-reported outcome measures were not available. Therefore, the magnitude of functional improvement could not be quantified using standardized scoring systems, and external comparability is limited. Future studies incorporating validated PROMs would provide a more comprehensive assessment of functional outcomes.

Third, multiple interventions were combined in this case, including nonunion-site refreshment, autologous cancellous bone grafting, circular external fixation, distraction osteogenesis, prolonged weight-bearing during external fixation, PTB orthosis after frame removal, rehabilitation, and adjunctive LIPUS. Therefore, it is not possible to determine the relative contribution of each individual component to the final outcome.

Fourth, the concept of reduced biological healing potential in congenital fibular deficiency was inferred from the clinical course of this case and from complication patterns reported in previous studies, rather than from direct biological, histological, or molecular evaluation in this patient [7,8,9]. Accordingly, this interpretation should be regarded as hypothesis-generating rather than definitive.

Finally, although LIPUS was used as an adjunctive treatment, current evidence regarding its effectiveness remains heterogeneous and controversial. Its specific contribution in this case should therefore be interpreted with caution [12,13,14].

## 5. Conclusions

Congenital fibular deficiency may present reconstructive challenges beyond deformity correction alone. In selected patients with chronic nonunion after previous treatment, durable limb salvage may be achieved through an individualized strategy addressing both biological and mechanical factors. This case highlights the importance of careful reconstructive planning and long-term follow-up after limb salvage reconstruction.

## Data Availability

All relevant data supporting the findings of this case report are included in the manuscript. Further details are available from the corresponding author upon reasonable request, subject to protection of patient privacy.
